# Race and sex differences in HDL peroxide content among American adults with and without type 2 diabetes

**DOI:** 10.1186/s12944-021-01608-4

**Published:** 2022-02-06

**Authors:** Shelby M. Flaherty, Elizabeth K. Wood, Carol D. Ryff, Gayle D. Love, Theodoros Kelesidis, Loni Berkowitz, Guadalupe Echeverría, Katherine Rivera, Attilio Rigotti, Christopher L. Coe

**Affiliations:** 1 Harlow Center for Biological Psychology, University of Wisconsin-Madison, Madison, WI USA; 2 Oregon Health and Science University, Portland, OR USA; 3 Institute on Aging, University of Wisconsin-Madison, Madison, WI USA; 4 Department of Medicine, University of California, Los Angeles, CA USA; 5 Departamento de Nutrición, Diabetes y Metabolismo; Escuela de Medicina, Pontificia Universidad Católica, Santiago, Chile

**Keywords:** Cholesterol, Dyslipidemia lipid peroxidation, Obesity, HDL function, Diabetes, Race

## Abstract

**Background:**

High-density lipoprotein (HDL) plays a critical role in protection against atherosclerosic and cardiovascular disease (ASCVD). In addition to contributing to clearing excess vascular cholesterol, HDL particles exhibit antioxidative functions, helping to attenuate adverse effects of oxidized low-density lipoproteins. However, these beneficial properties can be undermined by oxidative stress, inflammation, and unhealthy lifestyles and diet, as well as influenced by race and sex. Thus, when assessing cardiovascular risk, it is important to consider multifactorial aspects of HDL, including antioxidant activity rather than just total amount and type of HDL-cholesterol (HDL-C) particles. Because prior research showed HDL peroxide content (HDLperox) can be inversely associated with normal anti-oxidant HDL activity, elevated HDLperox may serve as a bioindicator of HDL dysfunction.

**Methods:**

In this study, data from a large national cohort of Americans was utilized to determine the impact of sex, race, and diabetes status on HDLperox in middle-aged and older adults. A previously developed cell-free fluorometric method was utilized to quantify HDLperox in serum depleted of apo-B containing lipoproteins.

**Results:**

In keeping with predictions, white men and diabetics exhibited HDLperox in the atypical upper range, suggestive of less functional HDL. White men had higher HDLperox levels than African American males (13.46 ± 6.10 vs. 10.88 ± 5.81, *p* < .001). There was also a significant main effect of type 2 diabetes (F(1,1901) = 14.9, *p* < .0001). Overall, African Americans evinced lower HDLperox levels, despite more obesity (10.3 ± 4.7 vs.11.81 ± 5.66 for Whites) suggesting that other aspects of lipid metabolism and psychosocial factors account for the higher prevalence of ASCVD in African Americans.

**Conclusion:**

This research helps to provide a more comprehensive understanding of HDL function in a racially and metabolically diverse adult population. HDLperox content was significantly different in adults with type 2 diabetes, and distinctive in nondiabetic White males, and suggests other processes account for the higher prevalence of ASCVD among African Americans.

**Supplementary Information:**

The online version contains supplementary material available at 10.1186/s12944-021-01608-4.

## Background

Although elevated levels of low-density lipoprotein-cholesterol (LDL-C) were historically considered to be the primary risk factor for atherosclerotic and cardiovascular disease (ASCVD), it is evident that low levels of high-density lipoprotein-cholesterol (HDL-C) are an equivalent concern [1, 2]. The negative association between low HDL-C and ASCVD risk reflects several important atheroprotective functions of high-density lipoproteins (HDL). Notably, assisting in reverse cholesterol transport (RCT), which enables removal of excess cholesterol from the vascular wall by transporting it to the liver for biliary secretion [3]. In addition, other beneficial functions of HDL include anti-oxidant, anti-inflammatory and anti-thrombotic activities, which can lessen the adverse effects of oxidized LDL-C and reduce foam cell and plaque formation [4, 5]. HDL is thought to perform anti-oxidant functions through peroxidase activity by its protein components, most likely paraoxonase 1 (PON-1), as well as apolipoproteinA-1 (apoA-1). Dietary supplements with high levels of phenolic flavonoids, including anthocyanines have been shown to increase HDL-C, HDL-associated PON-1, and improve chosterol efflux capacity, which would support cardioprotective functions [6]. However, a consensus on all of the specific pathways underlying atherogenic benefits of HDL has not been reached [7]. Moreover, the health-promoting effects of just modifying lipid levels without addressing HDL constituents may not be as protective as previously assumed, especially among those with metabolic and proinflammatory conditions such as type 2 diabetes [8–10]. Therapeutic modalities need to not only target increasing HDL-C levels, but should address the structural modifications that alter function, especially in dyslipidemic individuals [11, 12]. Specific aspects of HDL function also need more systematic investigation in larger population based studies of health.

Quantifying total HDL-C levels, as commonly done in clinical practice, provides information about the size of the HDL pool, but not about HDL composition or function. Methodological refinements in ex vivo assays that can specifically quantify lipid and non-lipid content of HDL may provide useful surrogate indicators of HDL function and anti-oxidant activity in cell-free biochemical conditions [13]. Prior research showed that high HDL peroxide content, when determined for a standardized amount of HDL-C, is associated with abnormal HDL function. In the context of oxidative stress, HDL has a higher load of lipid peroxides, which can decrease its protective activity against LDL oxidation. Specifically, reduced HDL function occurs in individuals with dyslipidemia, fatty liver disease and type 2 diabetes. It is also a significant side effect of the anti-viral medications used to treat HIV-infected individuals, and continues to be a concern even when there is an undetectable viral load [14]. In addition, the dyslipidemia associated with diabetes can affect not only the overall lipid levels in circulation, but can induce structural changes in HDL, altering its protein content and disrupting function [15, 16]. However, most of these studies have been performed with relatively small sample sizes that limit generalization to large populations and the previous research was not designed to detect race-related and sex differences.

A cell-free fluorometric method was employed that measures HDL associated lipid peroxidation (HDLperox), and offers a rapid and reproducible means to indirectly evaluate this apect of HDL function. It was applied on a large scale to determine the peroxide content of HDL-C among middle-aged and older Americans in the Midlife in the United States (MIDUS) project [17, 18]. Given the known racial differences in the prevalence of diabetes and ASCVD, this analysis offered an opportunity to compare HDL dysfunction in African Americans to White Americans descended from European family backgrounds. In general, most studies have found that African Americans are more likely to become diabetic and suffer from higher cardiovascular morbidity and mortality when compared to White Americans with similar clinical profiles [19]. This risk has been found to occur even when African Americans appear to have a more favorable lipid profile [20, 21]. In addition to racial differences, sex and glucoregulation are important to consider as potential modulators of HDL functionality. Previous research has documented significant differences in HDL-C levels and HDL function between men and women [22, 23]. When investigating the effects of race and sex on HDL functionality, it is also critical to consider the potential contribution of obesity, given its high prevalence among adults [24]. Thus, a secondary aim of this study was to compare the differential influence of adiposity and insulin resistance on HDLperox among Whites and African Americans, and the likely relationship between type 2 diabetes and HDL function.

## Materials and methods

### Participants

Middle-aged and older Americans (*N* = 1903) were surveyed and provided biomarker information as part of the larger MIDUS longitudinal survey (http://www.midus.wisc.edu/data/timeline.php). From 2004 to 2006, 1255 American adults were recruited from the 48 continental states via random telephone dialing as part of a first cohort of biomarker subsamples within the larger MIDUS-2 national study (35–86 years old). The same methodology was used for the MIDUS-Refresher (MIDUS-R) cohort between 2011 and 2016, during which 863 American adults were recruited (25–74 years old). To increase the racial diversity during MIDUS-2 and MIDUS-R, a further subsample was recruited in each cohort from Milwaukee, WI (MIDUS-2, *N* = 201; MIDUS-R, *n* = 117). Participants in the biomarker subsample were comparable to the larger national MIDUS survey with respect to age, sex, and marital status. However, participants in the biomarker project tended to be more educated than those participating only in the larger survey (25% attained a high school education, and 50% had completed some college) [17]. Although participation remained predominantly white (80.6%), there were a total of 370 African Americans in the two cohorts. Female and male participation was balanced overall (54.8% female), but there was a higher percentage of women among the older African American participants (descriptive statistics provided in Table 1).

**Table 1:** Demographic and biomarker variables for MIDUS participants stratified by race

	White 80.6% *N* = 1533	African American 19.4% *N* = 370	*X*^2^
**Demographic Variables**			
Sex^a^			
Male	48.5% (743)	31.6% (117)	**<.001**
Female	51.5% (790)	68.4% (253)	
Age^b^			
< 50	42.03 ± 5.48 (468)	41.45 ± 5.96 (163)	**<.001**
≥ 50	63.26 ± 8.7 (1065)	59.24 ± 7.18 (207)	
**Health and Biomarker Variables**			
Adiposity and Glucoregulation^c^			
BMI (kg/m^2^)	29.12 ± 6.16	33.02 ± 8.57	**<.001**
Waist circumference (cm)	96.88 ± 17.43	101.66 ± 18.43	**<.001**
HbA1c (%)	5.81 ± 0.85	6.41 ± 1.74	**<.001**
HOMA-IR	3.79 ± 5.80	5.22 ± 5.48	**<.001**
Blood Lipid Values^d^			
Total Cholesterol (mg/dL)	184.5 ± 38.3	182.7 ± 43.3	0.231
Triglycerides (mg/dL)	125.19 ± 71.54	110.64 ± 68.62	**<.001**
LDL-C (mg/dL)	102.48 ± 34.21	100.80 ± 37.78	0.187
Non-HDL-C (mg/dL)	127.9 ± 38.7	123.3 ± 43.0	**0.010**
HDL-C (mg/dL)	56.62 ± 18.78	59.42 ± 18.91	**0.004**
HDLperox	11.81 ± 5.66	10.3 ± 4.7	**<.001**

^a^ A higher percent of both White and Afr-Amer participants were female. ^b^ Although the age distribution was similar across race, more of the older participants were White. ^c^Afr-Amers were more likely to be overweight with more insulin resistance indicative of poor glucoregulation. ^d^ Afr-Amer tended to have more HDL-C. Even when HDL concentrations were controlled in the assay, the Afr-Amer particiants had significantly lower HDLperox levels

Biomedical assessments took place in Clinical Translational Research Centers (CTRC) at three participating universities. Specimen collection and testing were approved by the Health Sciences Institutional Review Board at the University of Wisconsin-Madison, as well as by the Institutional Review Boards at the University of California-Los Angeles and Georgetown University in Washington DC.

#### Sociodemographic variables

Age, race, and sex were obtained for all participants. Age was treated both as a parametric variable as well as categorized into two groups consisting of younger and older adults (< 50 and ≥ 50 years of age). Participants self-identified race (i.e., by asking the race with which each participant identified and the background races of their family) and were coded as White or African American. Many African-American participants were specifically recruited from neighborhoods in Milwaukee with predominantly African-American families. Sex was coded as female or male.

#### Anthropometric measures

Height, weight, and waist circumference were recorded by a nurse at each CTRC and used to calculate body mass index (BMI, weight/height, kg/m^2^). Poor glucoregulation for each participant was determined by meeting at least one of the three following criteria: ≥6.5% HbA1c, a diagnosis of diabetes by a physician, or use of prescribed medications for diabetes. The Homeostatic Model Assessment of Insulin Resistance (HOMA-IR) was calculated from insulin and glucose levels determined from fasted blood collected in a different vacuainer on the same day as the specimen used for assaying HDL-C. HOMA-IR was one of many variables log-transformed prior to analysis to ensure a normal distribution.

#### Blood collection and clinical lab results

12-h fasted blood (50 mL) was obtained between 0500 and 0700, with a modal time of 0605. The blood was centrifuged, and plasma and sera cryopreserved for later testing. In addition, whole blood was collected in a separate vacutainer to determine HbA1c as an integrated measure of glucose levels over the prior several weeks. Blood was either transported locally or shipped cool via overnight carrier for analysis at the same diagnostic laboratory via turbidimetric immunoinhibition assay (Meriter Labs, Madison, WI). A traditional lipid panel was also determined at this Clinical Laboratory Approvement Amendments (CLIA) certified laboratory. It included total cholesterol, HDL-C, LDL-C, and triglyceride levels (mg/dL). LDL-C level was calculated using the following formula: LDL = total cholesterol - HDL-C - triglyceride/5. When triglycerides were lower than 400 mg/dL, LDL-C level was calculated using the following formula: LDL-C (mg/dL) = Total cholesterol (mg/dL) - HDL-C (mg/dL) – triglycerides (mg/dL)/5.

There were 3 measures of glucoregulation: HbA1c (%) and fasted insulin and glucose, from which HOMA-IR was calculated. Type 2 diabetes was determined by meeting at least one of the 3 following criteria: ≥6.5% HbA1c, a diagnosic of diabetes by a physician, or use of medications prescribed for diabetes.

#### Biochemical assay of HDLperox

HDL samples were prepared by depleting apoB containing particles from serum via a precipitation method using dextran sulfate (Sigma) and magnesium chloride (Sigma) at concentrations of 2 μM and 50 mM, respectively [24]. A fixed volume protocol for analysis of lipid peroxidation was adapted from a previously developed fluorescence protocol [13]. Adaptations to optimize this assay that differ from the published protocol include the use of polypropylene round bottom plates (Greiner), and inclusion of a positive control of purified HDL-C (Lee Biosolutions). Control HDL-C was maintained in stock concentration of 2070 mg cholesterol/dL, which was diluted to a working concentration of 300 mg/dL using 0.15 NaCl for each assay. Both reagent preparations and the fluorescence-based HDLperox assay were conducted in a darkened room to minimize light exposure and extraneous oxidation. Sera were assayed in triplicate on one plate. Fluorescence was measured with a Synergy H1 fluorimeter (Biotek) at 530 nm and 590 nm wavelength. Quantification of HDL-C in all sera, and for the positive control of purified HDL-C, was performed with the WAKO Cholesterol E kit (Fujifilm), using a microplate reader (MRX^e^ DYNEX Magellan Biosciences) as well as at the Meriter clinical laboratory.

The following calculations were employed to generate the final HDL peroxide content used in the analyses:

1. Fluorescence in sample – Fluorescence in blank = Peroxidized HDL sample in flurorescence units (FU).
2. FU/ HDL-C in sample (mg/dL) = Normalized peroxidized HDL per 1 mg/dL HDL-C (FU/mg HDL-C), which was the basic unit of measure for this assay.
3. Normalized peroxidized HDL per 1 mg/dL HDL-C in sample (FU per mg/dL HDL-C)/ standardized peroxidized HDL in purified HDL reference control = HDLperox*

*Steps 2 and 3 were performed for HDL-C, which was measured both with the WAKO kit and by the clinical laboratory, and then averaged. The same pool of purified HDL-C was included in each assay to provide a standard and common reference point for harmonizing results across the assays needed to test 1903 sera.

### Statistical analyses

Descriptive summary statistics were generated to examine means, variance, data distribution, and to identify outlier values. BMI, HbA1c, HOMA-IR, HDL-C, LDL-C, non-HDL-C, waist circumference, and triglycerides were log_e_ transformed to normalize their distributions. All other variables were mean-centered. Chi-square and t-tests were used to compare sociodemographic and health variables between Whites and African Americans. Two-way ANOVA was used to compare differences in HDLperox by race and sex. Three-way ANOVA was used to compare differences in HDLperox by race, sex, and diabetes status. Tukey’s honest significance tests were performed for post hoc contrasts to determine pairwise significance. Multivariate linear regression models examined the association between HDLperox and health variables, adjusting for sex and diabetes status. In addition, the influence of race on HDLperox was considered in a separate hierarchal regression model, adjusting for sex (male as reference), diabetes status (nondiabetic as reference), as well as considering the effects of age, obesity, and non-HDL-C. Interactions of diabetic status with race and sex were evaluated, but are not reported because none were statistically significant. The influence of dyslipidemia, insulin resistance (HOMA-IR), and obesity (waist circumference) were considered independently as possible mediators. The possible influence of prescription medication was considered in two ways. First, self-reported use of any of 23 different drugs was included as a categorical covariate in the hierarchical regression model. Second, the specific influence of lipid-lowering medications and supplements on the differences in HDLperox in diabetics and between women and men was analyzed (focusing on antihyperlipidemic agents, cholesterol absorption inhibitors, HMC-COA reductase inhibitors, bile acid sequestrants, fibrates, niacin, omega-3). All tests were two-tailed, and alpha threshold set at *p* < .05. Statistical analyses were conducted with SPSS (version 25) and R-studio Desktop (version 3.2.5) software.

## Results

### Descriptive statistics

Descriptive statistics and results from chi-square and t tests for all demographic and biological variables are provided in Table 1. On average, the African American participants exhibited healthier lipid profiles when compared to Whites. African Americans were more likely to have higher HDL-C (African Americans: 59.4 ± 18.9 mg/dL vs Whites: 56.6 ± 18.8 mg/dL; *p* = .01) and lower triglycerides (African Americans: 110.6 ± 68.6 mg/dL vs. Whites: 125.2 ± 71.5 mg/dL; *p* < .001) and lower non-HDL-C (African Americans: 110.6 ± 68.6 mg/dL vs. Whites: 125.2 ± 71.5; *p* < .001) when compared to Whites. This race difference was evident in spite of the fact that more African Americans evinced signs of insulin resistance (HOMA-IR: African Americans: 5.2 ± 5.5 vs. Whites: 3.8 ± 5.8; *p* < .001) and had higher HbA1c values (African Americans: 6.4 ± 1.7% vs. Whites: 5.8 ± 0.8%; *p* < .001). Type 2 diabetes was prevalent overall, but higher among African Americans (33.8%) when compared to White adults (13.8%). African Americans were also more likely to be overweight as reflected by a higher BMI (African Americans: 33.0 ± 8.6 kg/m^2^ vs. Whites: 29.1 ± 6.2 kg/m^2^; *p* < .001). On average, men had lower HDL-C levels (men: 50.4 ± 16.5 mg/dL vs. women: 62.8 ± 18.8 mg/dL; *p* < .001), higher triglycerides (men: 135 ± 75.4 mg/dL vs. women: 111.9 ± 65.8 mg/dL; *p* < .001) and exhibited a small but significant difference in non-HDL-C when compared to women (men: 128.0 ± 40.3; women: 126.1 ± 39.1 mg/dL; *p* < .001).

White men exhibited the highest HDLperox when compared with white women or African Americans (white men: 13.5 ± 6.1 vs. white women: 10.6 ± 4.7; *p* < .001; African American men: 10.9 ± 5.8; *p* < .001; African American women: 10.0 ± 5.8; *p* < .001). Table 2 provides the summary statistics on HDLperox stratified by race, sex, and diabetic status.

**Table 2:** Means (S.D) for biomarker and clinical measures organized by race, sex, and diabetes status

	Men						Women					
	Nondiabetic			Diabetic			Nondiabetic			Diabetic		
Variable	White	Afr-Amer	*p*-value	White	Afr-Amer	*p*-value	White	Afr-Amer	*p*-value	White	Afr-Amer	*p*-value
*n*	629	85		114	32		692	160		98	93	
**Age (yrs.)**	56 ± 13^b^	50 ± 10^b^	**<.001**	64 ± 12	56 ± 10	**<.001**	55 ± 12^b^	49 ± 11^b^	**<.001**	59 ± 11	54 ± 11	**<.001**
< 50	42 ± 6	42 ± 6	**0.004**^**a**^	43 ± 6	45 ± 4	0.177^a^	42 ± 5	41 ± 6	**<.001**^**a**^	44 ± 3	42 ± 5	0.081^a^
≥ 50	63 ± 8^b^	58 ± 7		67 ± 9	60 ± 9		62 ± 9	59 ± 7		63 ± 8	60 ± 7	
**Adiposity and Glucoregulation**												
BMI (kg/m^2^)	29.1 ± 4.8^b^	29.1 ± 6.7^b^	**<.001**	31.7 ± 5.9	31.7 ± 6.7	**<.001**	28.0 ± 6.1^b^	33.1 ± 8.1^b^	**<.001**	33.7 ± 9.0	36.8 ± 9.8	**<.001**
Waist Circumference (cm)	102 ± 15^b^	98 ± 17^b^	**<.001**	111 ± 17	107 ± 18	**<.001**	89 ± 15^b^	98 ± 16^b^	**<.001**	104 ± 18	109 ± 22	**<.001**
HOMA-IR	3.5 ± 3.3^b^	3.7 ± 3.8^b^	**<.001**	9.4 ± 3.7	7.2 ± 6.9	**<.001**	2.7 ± 2.5^b^	4.4 ± 4.5^b^	**<.001**	7.2 ± 11.9	7.3 ± 6.9	0.286
HbA1c (%)	5.5 ± 0.42^b^	5.6 ± 0.52^b^	**<.001**	7.5 ± 1.4	7.7 ± 2.5	**<.001**	5.6 ± 0.4^b^	5.7 ± 0.4^b^	**<.001**	7.1 ± 1.3	8.0 ± 2.3	**<.001**
**Blood Lipid Values**												
Total Cholesterol (mg/dL)	180 ± 37^b^	188 ± 47^b^	**<.001**	164 ± 39	169 ± 47	**<.001**	192 ± 37^b^	180 ± 39	**<.001**	181 ± 42	187 ± 45	**<.001**
LDL-C (mg/dL)	102 ± 33^b^	106 ± 45	**<.001**	87 ± 36	93 ± 40	**<.001**	105 ± 34^b^	99 ± 33	**<.001**	97 ± 40	103 ± 37	**<.001**
Triglycerides (mg/dL)	132 ± 71^b^	118 ± 74	**<.001**	168 ± 89	128 ± 86	**<.001**	109 ± 62^b^	94 ± 56^b^	**<.001**	147 ± 84	126 ± 72	**<.001**
non-HDL-C (mg/dL)	130 ± 38^b^	130 ± 51	**<.001**	121 ± 40	119 ± 46	**<.001**	128 ± 39	118 ± 37	**<.001**	127 ± 45	128 ± 44	**<.001**
HDL-C (mg/dL)	51 ± 16^b^	58 ± 21^b^	**<.001**	42 ± 14	50 ± 14	**<.001**	65 ± 19^b^	62 ± 17	**<.001**	54 ± 18	59 ± 20	**<.001**
HDLperox	13.2 ± 6^b^	10.4 ± 6	**<.001**	14.9 ± 6	12.1 ± 5	**<.001**	10.1 ± 5^b^	9.7 ± 4	**<.001**	11.7 ± 5	10.5 ± 5	**<.001**

^a^chi-square test ^b^*t-*test comparing race and diabetes status Significance shown by bolded *p* value

### HDLperox

**Fig. 1:**
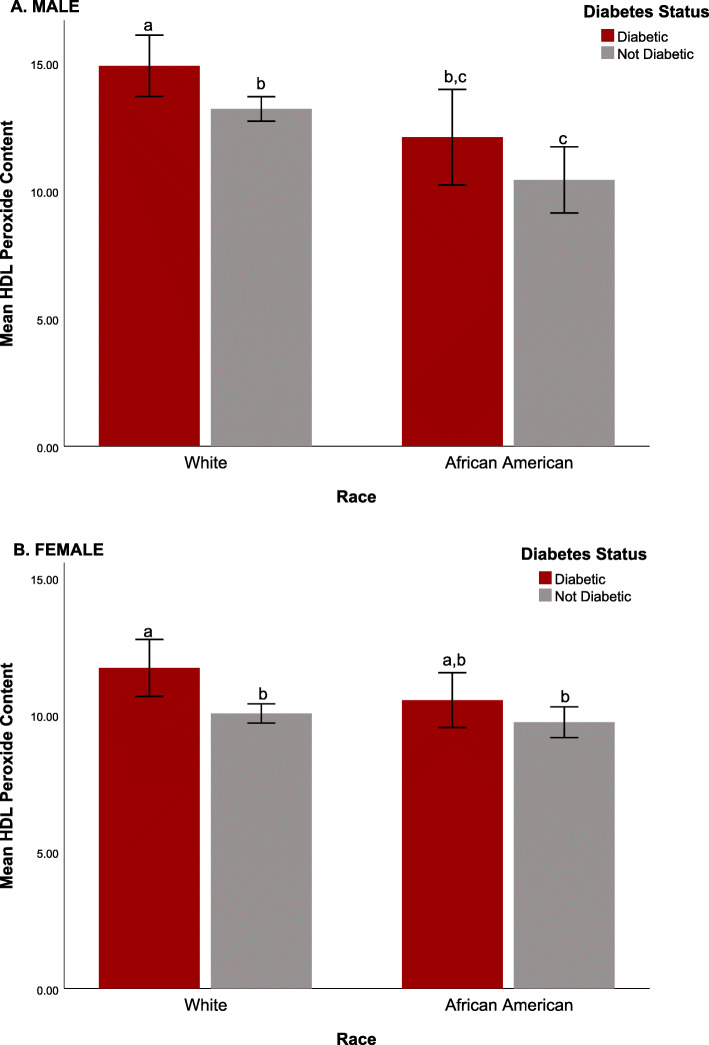
HDLperox in White and African American males and females shown with respect to diabetic status. Significantly more HDLperox was found in White males when compared to African American males. Diabetes increased HDLperox in White males and females, as well as African American males, but did not significantly increase HDLperox among African American women. Letters indicate significance *(p* < .05) based on a two factor ANOVA assessing the influence of race and diabetic status on HDLperox. Tukey post hoc tests of significance are indicated by *p < 0.05, **p < .01, and ***p < .001. Graphs show peroxidized HDLin serum (FU/HDL-C, mg/dL), standardized across assays by reference to purified HDLperox

**Fig. 2:**
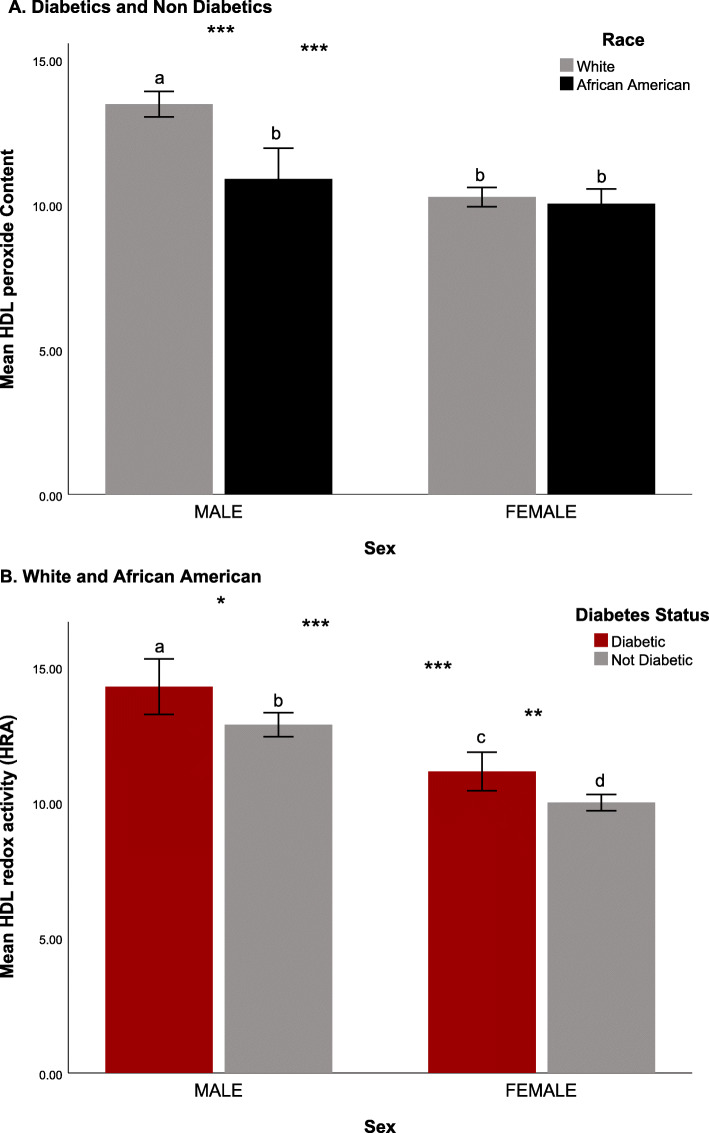
Significant influence of race, sex, and diabetes on HDLperox. White males had the highs HDLperox suggestive of poorer HDL function. Diabetics had higher HDLperox levels than nondiabetic adults. Significance indicated by letters. After attaining significance in a two-way ANOVA, the Tukey test was used for post hoc comparisons *p < 0.05, **p < .01, and ***p < .001

One-way ANOVA results indicated there was a significant main effect of race on HDLperox (F (1,1901) = 31.3, *p* < .0001), which was not congruent with the overall higher occurrence of obesity and diabetes in the African American participants. HDLperox in the African Americans was lower than in the White adults (African Americans: 10.3 ± 4.7 vs. Whites: 11.81 ± 5.66; *p* < .001). When probing this racial difference further by considering the dual influence of race and sex (F (1,1899) = 20.3, *p* < .0001) it was evident that the significant racial differences in HDLperox were driven primarily by the difference between African American and White men (see Fig. 1A). White men had higher HDLperox (white men: 13.46 ± 6.10 vs. African American men: 10.88 ± 5.81, *p* < .001) suggesting that some anti-oxidant functions of HDL might be impaired. In contrast, a pairwise comparison of HDLperox in White and African American women did not indicate there was a similar racial difference (White females: 10.26 ± 4.73 vs. African American females: 10.03 ± 4.07, *p* = .520; see Fig. 2A). There was also a significant main effect of diabetes (F (1,1901) = 14.9, *p* < .0001; see Fig. 2B), which affected HDLperox in both racial groups, although it was not as pronounced in African American women (see Fig. 1B). The expected negative effect of diabetes on HDLperox was evident in both middle-aged and older adults. While there was a trend for an influence of age in the overall models, a separate test with age considered as a categorical variable (i.e., under and over 50 years of age) indicated the difference in HDLperox between middle-aged and older adults was lessened after controlling for the influence of sex and race. The potential association between HDLperox and selected variables reported by many studies to contribute to cardiovascular risk was evaluated further in several additional analyses. As shown in Table 3, after adjusting for covariates (including sex, age, and diabetes), a linear regression model showed that HDLperox was significantly associated with several risk factors for ASCVD, including obesity (BMI, waist circumference), insulin resistance (HOMA-IR), and the traditional clinical measures of lipid metabolism (non-HDL-C, LDL-C and triglycerides) in both African Americans and Whites. HDLperox was inversely associated with the circulating levels of HDL-C in both racial groups. The analysis indicated that an individual with higher HDL-C levels would have lower HDLperox; specifically, for each 1 mg/dL increase in HDL-C in circulation, HDLperox is predicted to be 19.5% percent lower.

**Table 3:** Linear regression between HDLperox and health variables across race, controlling for demographic variables and diabetes in the MIDUS participants

Variable	White		African American	
	*β* (SE)	*p*-value	*β* (SE)	*p*-value
**Adiposity and Glucoregulation**				
BMI (kg/m^2^)	.445 (.048)	**<.001**	.433 (.083)	**<.001**
HOMA-IR	.141 (.012)	**<.001**	.157 (.023)	**<.001**
Waist Circumference (cm)	−.023 (.077)	.764	.117 (.100)	0.242
**Blood Lipid values (mg/dL)**				
Total Cholesterol	−.146 (.047)	**.002**	−.181 (.092)	0.051
LDL-C	.065 (.027)	**.016**	.109 (.053)	**0.041**
Triglycerides	.260 (.018)	**<.001**	.286 (.038)	**<.001**
Non-HDL-C	.224 (.031)	**<.001**	.245 (.060)	**<.001**
HDL-C	−.612 (.025)	**<.001**	−.893 (.052)	**<.001**

*Note.* All variables were loge transformed for this analysis

White and African Americans evinced similar relationships between adiposity and the glucoregulatory measures, as well as similar associations between blood lipids and HDLperox

**Fig. 3:**
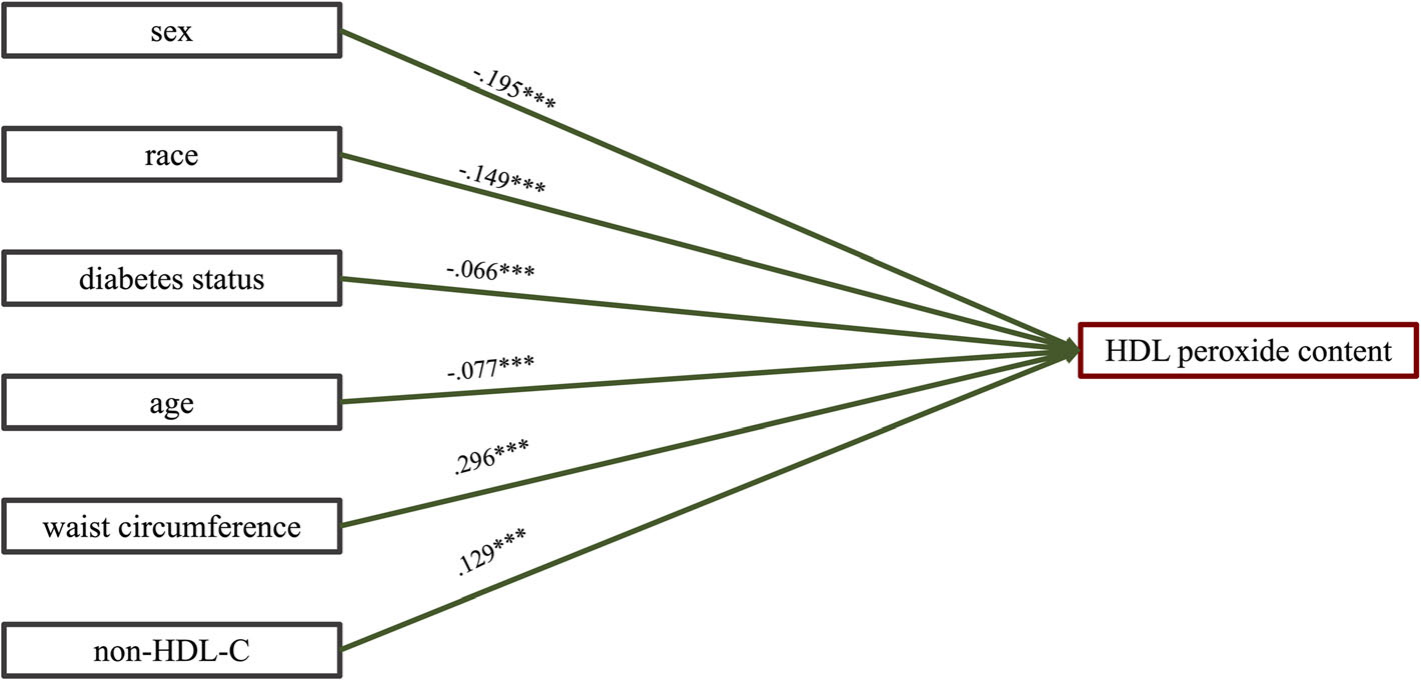
Hierarchical regression analysis of variables influencing HDLperox (*N* = 1903). Overall model: *F* (6,1894) = 78.47, *p* < .0001; R^2^ = .199, Adjusted R^2^ = .197. Standardized coefficients are shown. The model indicated that being male, white, having diabetes, a younger age, larger waist circumference, and higher non-HDL-C was predictive of HDL peroxide content. This model accounted for 19.9% of HDL perox variance. Significance indicated by asterisks (*p < .05, **p < .01, ***p < .001). Results from 6 sequential models are presented in Suppl Table 1

In order to evaluate other factors that could account for the observed sex and racial differences in HDLperox, a series of hierarchical regression models were run, sequentially adjusting for relevant covariates. Self-reported use of any of 23 different prescription medications was included as a covariate. Statistical results from this series of 6 models are presented in Supplemental Table S1; Fig. 3 provides a visual summary of the final model. Significant differences in HDLperox between men and women were retained in all models, as was the difference in HDLperox between African Americans and Whites, even when accounting for age, obesity and diabetes status. Notably, the significant associations between sex (i.e., being male) and race (i.e., being White) and higher HDLperox remained, even after accounting for non-HDL-C, the increasingly used clinical index of atherogenic dyslipidemia. The final model yielded a statistically significant R^2^ of 0.199 (*p* < .0001).

With age treated as a categorical variable (< 50 yrs. vs. ≥50 yrs.), there was a significant effect of age. However, it was because HDLperox content was higher in middle-aged adults, suggestive of reduced HDL functional capacity in many of the younger participants. This model was also confirmatory of the previous analyses indicating that the White males had the highest HDLperox levels, even after accounting for the effect of the participant age.

### Exploratory analysis of lipid-lowering medications

Even though self-reported use of many prescription medications had been considered as a covariate in the hierarchical regression model, the possibility of a specific, moderating effect of just lipid-lowering drugs and supplements on the observed effects of sex, race and diabetes was then assessed (i.e., antihyperlipidemic agents, cholesterol absorption inhibitors, HMC-COA reductase inhibitors, bile acid sequestrants, fibrates, niacin, omega-3). Among the White participants, 369 of 1533 (24%) were taking one of these drugs, but the sex difference with higher HDLperox in men continued to be evident in both medicated and nonmedicated males (see Table S2). Similarly, the lower HDLperox in African Americans was not attributable to taking more lipid-lowering medications (see Table S3). In the MIDUS cohort, 82% of the African Americans, and 75% of the White Americans reported that they were not taking lipid-lowering medications. Lower HDLperox was found in both African Americans taking medications and those who were not. Finally, an ANOVA comparing the difference in HDLperox between nondiabetic and diabetic participants indicated that the use of lipid-lowering medications did not lessen the influence of diabetes on HDLperox (Table S4). Among participants meeting study criteria for type 2 diabetes, 207 (61%) reported they were not taking a lipid-lowering medication.

## Discussion

To our knowledge, this study is the first to assess lipoperoxide levels in HDL among middle-aged and older American adults in such a large scale population-based manner. It should be acknowledged that this type of ex vivo determination of HDLperox provides an indirect assessment of HDL anti-oxidative potential, and is not a direct measure of its in vivo function. However, the assay does offer some insight into how HDL particles and associated lipids would likely respond and function in the presence of reactive oxygen species.

We identified significant differences in HDLperox between women and men, which would usually be interpreted to indicate a gender difference in the anti-oxidative functions in HDL. There was also a racial difference between White participants and African Americans. The difference between White men and women was in the expected direction and is consistent with the well-documented sex differences in lipid metabolism and risk for ASCVD [22, 23]. In contrast, the effect of race was not anticipated and does not seem to be congruent with the higher morbidity and mortality from ASCVD among African Americans [20]. Based on our results, the HDLperox in African American adults was on average 12.7% lower than the HDLperox in White Americans. This racial distinction was especially striking for African American men, given the high HDLperox in White men. Whether these differences in HDLperox will prove to be correlated with other oxidative biomarkers and circulating levels of anti-oxidants in the MIDUS cohort is currently being determined.

The direction and magnitude of the observed race effect is especially noteworthy given that the prevalence of type 2 diabetes among African American participants was twice that of the White participants. It is known that diabetes can decrease HDL-C and increase triglycerides, oxidative stress and inflammation, which would likely contribute to structural and functional changes in HDL and decreased RCT function [25]. Other research has indicated that there are significant differences in PONS-1 activity in diabetic individuals [16]. However, the interaction between diabetes and gender they reported was not the same as we observed, because diabetes appeared to have a much larger effect on PON-1 in diabetic women.

Rosta et al. concluded the impairment of PON-1 arylesterase activity in type 2 diabetes was gender-specific, with women affected more than men. However, some studies suggest that PON-1 activity as measured by the paraoxon substrate may more closely correlate with HDL function [26, 27]. Thus, differences between assay methods could explain some of the discrepancies in conclusons. Further research is needed to better resolve how gender affects HDL antioxidant function, with a simultaneous assessment of HDL peroxide content, PON-1 arylesterase activity and PON1 paraoxonase activity determined in the same sample.

In vitro studies suggest that oxidative modification of apoA-I may also impair HDL’s ability to mediate cholesterol efflux by inhibiting the remodeling/exchange of apoA-I. Consistent with the hypothesis that HDLperox is a relevant mediator in early atherogenesis, the increases in HDLperox that persist in HIV-infected individuals promote early instigators of atherogenesis in vitro, including monocyte/macrophage chemotaxis and monocyte-derived foam cell formation (MDFCF) [28]. In addition, in a prospective 1-year cohort study, adolescent males (17.4 ± 1.6 years) with severe obesity, were followed after weight loss achieved by vertical sleeve gastrectomy [29]. Their HDLperox was a better predictor of improvement in HDL function when compared to HDL cholesterol efflux capacity and HDL anti-oxidative capacity. Finally, there is some clinical evidence that the benefits of anti-oxidant activity generalize to other ethnic and national groups outside the US [30]. In the Mashhad Stroke and Heart Atherosclerotic Disorder (MASHAD) study of 330 Iranian adults, 35–65 years of age, who had a median follow-up period of 7 years, reduced antioxidant function independently predicted risk for CVD (odds ratio, 1.62; 95% confidence interval, 1.41–1.86; *p* < 0.001).

Within the MIDUS cohort, 33.8% of the African American adults met criteria for type 2 diabetes as compared to 13.8% of the white adults. This racial disparity in glucoregulation was confirmed by HOMA-IR measured from the same fasted blood samples. The prevalence of obesity and type 2 diabetes was especially high among African American women; yet their sex and race appeared to be associated with some protection lessening or potentially buffering the negative influence of their diabetes. This interpretation concurs with previous research that found African American women had higher triglycerides and CVD than white women, while also having higher HDL-C and a higher concentration of apoA-1 [31]. At the same time, progression to type 2 diabetes would create a dysregulated environment within the body that decreases HDL-C, increasing triglycerides and oxidative stress and inflammation, which would then be likely to cause structural and functional changes in HDL. Therefore, further research is now needed to determine the mechanism accounting for this protection in African American women, given that HDL more commonly becomes dysfunctional in diabetic individuals. Proinflammatory processes were also found to result in oxidized and dysfunctional HDL in septic patients, and persistence of altered HDL was associated with poorer clinical outcomes [27].

Finally, it was also of interest that the sex and race differences were evident in both middle-aged and older adults, given that there are also age-related increases in dyslipidemia and more abnormal triglyceride metabolism [32, 33]. A statistically significant effect of age was apparent primarily in the hierarchical regression models, which considered the contribution of each predictor variable separately. But it was driven primarily by the higher HDLperox in middle-aged white men. This finding is suggestive of poorer lipid health among many middle-aged participants, which likely reflects secular trends over the last several decades. Younger Americans are becoming heavier and less healthy in middle adulthood. In addition, some of the oldest adults participating in MIDUS may be a healthy and resilient subgroup of elderly individuals.

### Strengths and limitations

Notwithstanding that this study of middle-aged and older adults is the largest application of a HDLperox assay to date, and a number of unique findings were generated, there were also limitations. It should be acknowledged that the origins and consequences of gender and race-related differences in physiology and disease are multifactorial [34]. The current analysis did not consider many lifestyle variables and health care practices that can influence lipid metabolism and function, including diet, smoking, alcohol consumption and physical activity. In addition, while the MIDUS project was initiated as a nationally representative survey with participants from all 48 continental states, the recruitment of a racially diverse sample was achieved via oversampling African Americans from a single city (Milwaukee, WI). This recruitment approach was chosen to facilitate participation in the biomarker project because of the proximity to the CTRC site in Madison, WI. It created the opportunity to consider how race might influence HDLperox in the context of type 2 diabetes. The rigorous protocol and standardization of the HDLperox assays while conducting nearly 2000 tests was another strength of the project. But it should be acknowledged that our HDL assay focused on a single endpoint. Interpretation and implications for functional activity would have been enhanced by the inclusion of additional measures, including PON-1 activity [35, 36].

## Conclusion

In conclusion, this survey of American adults identified both racial and sex influences on HDLperox. The higher HDLperox content in White males was striking when contrasted with white women and also with the lower HDLperox in African Americans of both sexes. While the effect of type 2 diabetes on HDLperox was in the predicted direction, the findings underscore the many health and policy concerns engendered by the rising prevalence of obesity and diabetes worldwide [37, 38]. A better understanding of the factors associated with the risk for ASCVD is a pressing healthcare issue. Finally our findings on HDLperox in African Americans suggest that other mediators and psychosocial factors likely account for the well-established racial disparities in cardiovascular health. Nevertheless, the relative ease of employing the HDLperox assay on cryopreserved serum samples makes it a sensitive and useful biomarker for investigating how HDL responds to inflammatory conditions and metabolic disorders, and the role it likely plays in dysfunctional HDL antioxidant activity.

## Supplementary Information

**Additional file 1.**

## Data Availability

In addition to the publicly available data at ICPSR, the MIDUS Portal offers researchers access to rich searchable variable-level metadata, longitudinal harmonization information, and the ability to download customized datasets and codebooks. http://midus.wisc.edu/data/index.phphttps://midus.colectica.org/.

## Abbreviations

LDL-C: Low-density lipoprotein cholesterol.
CVD: Cardiovascular disease.
HDL-C: High-density lipoprotein cholesterol.
HDL: High-density lipoprotein.
RCT: Reverse cholesterol transport.
HDLperox: HDL peroxide content.
PON-1: Paraoxonase 1.
apoA-1: Apolipoprotein A1.
MIDUS: Midlife in the United States.
CTRC: Clinical Translational Research Center.
CLIA: Clinical Laboratory Improvement Amendments.
apoB: Apolipoprotein B.
HOMA-IR: Homeostasis model assessment-estimated insulin resistance.
MASHAD: Mashhad stroke and heart atherosclerotic disorder.
MDFCF: Monocyte-derived foam cell formation.
