# Supplementary material for: Race and sex differences in HDL peroxide content among American adults with and without type 2 diabetes
Source: Lipids Health Dis. 2022 Feb 6;21:18. doi: 10.1186/s12944-021-01608-4 (PMC8818198; doi:10.1186/s12944-021-01608-4)
Supplement: Supplementary file 1 — Additional file 1. [file 12944_2021_1608_MOESM1_ESM.zip › Suppl Table 2-4.pdf]

Supplemental Tables S2-S4 provide the number of participants taking lipid-lowering medications or supplements, which could have lipid-lowering actions (i.e., antihyperlipidemic agents, cholesterol absorption inhibitors, HMC-COA reductase inhibitors, bile acid sequestrants, fibrates, niacin, omega-3). Mean HDLperox content found for each subgroup is also shown. The overall pattern of findings was not changed by considering these medications. There were sufficient participants not taking these medications to discern similar effects of sex, diabetes and race on HDLperox content.

Table S2. Significant sex difference in HDLperox content found only in White participants, The male/female was similarly evident in those taking lipid-lowering drugs and supplements.

	Medication	No medication	Total
White men	13.38 (n = 235)	13.49 (n = 508)	n = 743
White women	10.79 (n = 134)	10.15 (n = 656)	n = 790
	n = 369	n = 1164	n = 1533

Table S3. Racial difference was also evident in those not taking lipid-lowering medications, , with lower HDLperox content in African-American participants, Percent of participants not taking these drugs or supplements was similar in both racial groups (82% AA and 72% White participants, respectively).

	Medication	No Medication	Total
White	12.44 (n = 369)	11.61 (n = 1164)	n = 1533
AA	11.31 (n = 66)	10.08 (n = 304)	n = 370
	n = 435	n = 1468	n = 1903

Table S4. Higher HDLperox content was found in diabetic adults, including in those taking lipid-lowering medications and among those who were not. Among the diabetic adults, 62% were not taking lipid-lowering medications. The latter subgroup allowed us to determine HDLperox content in 207 diabetic adults without the influence of these medications.

	Medication	No Medication	Total
Non-diabetic	11.96 (n = 305)	11.14 (n = 1261)	n = 1566
Diabetic	12.98 (n = 130)	12.20 (n = 207)	n = 337
	n = 435	n = 1468	n = 1903
