# Supplementary material for: Race and sex differences in HDL peroxide content among American adults with and without type 2 diabetes
Source: Lipids Health Dis. 2022 Feb 6;21:18. doi: 10.1186/s12944-021-01608-4 (PMC8818198; doi:10.1186/s12944-021-01608-4)
Supplement: Supplementary file 1 — Additional file 1. [file 12944_2021_1608_MOESM1_ESM.zip › SupplT1.pdf]

Supplemental Table 1. Hierarchical regression analysis for variables predicting HDLperox *(n* = 1903)

	Model 1			Model 2			Model 3			Model 4			Model 5			Model 6		
**Variable**	B	SE	ß	B	SE	ß	B	SE	ß	B	SE	ß	B	SE	ß	B	SE	ß
Sex	-.246	.018	-.293***	-.236	.018	-.282***	-.235	.018	-.281***	-.237	.018	-.283***	-.158	.019	-.495***	-.163	.018	-.195***
Race				-.094	.023	-.089***	-.119	.024	-.113***	-.134	.024	-.127***	-.169	.023	-.160***	-.156	.023	-.149***
Diabetes							-.126	.024	-.116***	-.140	.025	-.129***	-.063	.024	-.057*	-.072	.024	-.066**
Age										-.002	.001	-.069**	-.003	.001	-.089***	-.003	.001	-.077***
Obesity													.704	.055	.296***	.640	.055	.269***
Non-HDL																.169	.027	.129***
R^2 (adj.)	.086 (.086)***			.094 (.093)***			.107 (.105)***			.111 (.109)**			.183 (.181)***			.199 (.197)***		
ΔR^2				.008			.013			.004			.072			.054		

Note. **p* < .05, ***p* < .01, ****p* < .001. Sex, race, and diabetes status were $\log_e$ transformed.
